# The Effects of Asprosin on Exercise-Intervention in Metabolic Diseases

**DOI:** 10.3389/fphys.2022.907358

**Published:** 2022-07-11

**Authors:** Lifei Liu, Yuhao Liu, Mei Huang, Miao Zhang, Chenyu Zhu, Xi Chen, Samuel Bennett, Jiake Xu, Jun Zou

**Affiliations:** ^1^ School of Kinesiology, Shanghai University of Sport, Shanghai, China; ^2^ Department of Rehabilitation, The People’s Hospital of Liaoning Province, Shenyang, China; ^3^ Department of Orthopaedic, The First Affiliated Hospital, Guangzhou University of Chinese Medicine, Guangzhou, China; ^4^ School of Biomedical Sciences, University of Western Australia, Perth, WA, Australia; ^5^ School of Sports Science, Wenzhou Medical University, Wenzhou, China

**Keywords:** asprosin, exercise, metabolism disease, T2DM, obesity, pcos

## Abstract

Fibrillin is the major constituent of extracellular microfibrils, which are distributed throughout connective tissues. Asprosin is derived from the C-terminal region of the FBN1 gene, which encodes profibrillin that undergoes cleavage by furin protein. In response to fasting with low dietary glucose, asprosin is released as a secreted factor from white adipose tissue, and is transported to the liver for the mediation of glucose release into the blood circulation. Through binding to OLFR734, an olfactory G-protein-coupled receptor in liver cells, asprosin induces a glucogenic effect to regulate glucose homeostasis. Bioinformatics analyses revealed that the FBN1 gene is abundantly expressed in human skeletal muscle-derived mesoangioblasts, osteoblast-like cells, and mesenchymal stem cells, indicating that the musculoskeletal system might play a role in the regulation of asprosin expression. Interestingly, recent studies suggest that asprosin is regulated by exercise. This timely review discusses the role of asprosin in metabolism, its receptor signalling, as well as the exercise regulation of asprosin. Collectively, asprosin may have a vital regulatory effect on the improvement of metabolic disorders such as diabetes mellitus and obesity via exercise.

## Introduction

Asprosin was initially identified as a new class of hormone, or a centrally acting orexigenic hormone that regulates the liver to release hepatic glucose and increase plasma glucose levels (Greenhill, 2016; Romere et al., 2016). It was subsequently revealed that asprosin acts as a central appetite stimulator, or a fasting-induced glucogenic protein hormone (Duerrschmid et al., 2017), via a signalling pathway similar to ghrelin, also known as lenomorelin or **“hunger hormone”** (Donma and Donma, 2018). Asprosin deficiency is found in patients with **neonatal progeroid syndrome (NPS)**, while excess production of asprosin is detected in the condition of insulin resistance and obesity (Greenhill, 2016; Romere et al., 2016). It is proposed that asprosin in cooperation with ghrelin is beneficial to cachexia-anorexia, a complex metabolic syndrome occurring in severe burns victims (Donma and Donma, 2018). These findings imply that asprosin plays an essential role in a range of metabolic-related diseases. Exercise improves the outcomes of metabolic disorders, such as promoting energy substrate redistribution, losing fat mass, and reducing inflammation (Gonzalez-Gil and Elizondo-Montemayor, 2020). Notably, exercise affects the release of asprosin, which may regulate the corresponding metabolism (Ko et al., 2019). In this review, we summarize the expression profile of asprosin and the effects of exercise on asprosin to mediate metabolic diseases, such as diabetes mellitus (DM), obesity and polycystic ovary syndrome (PCOS).

## Molecular Structure, Expression and Function of Asprosin

**FIGURE 1:**
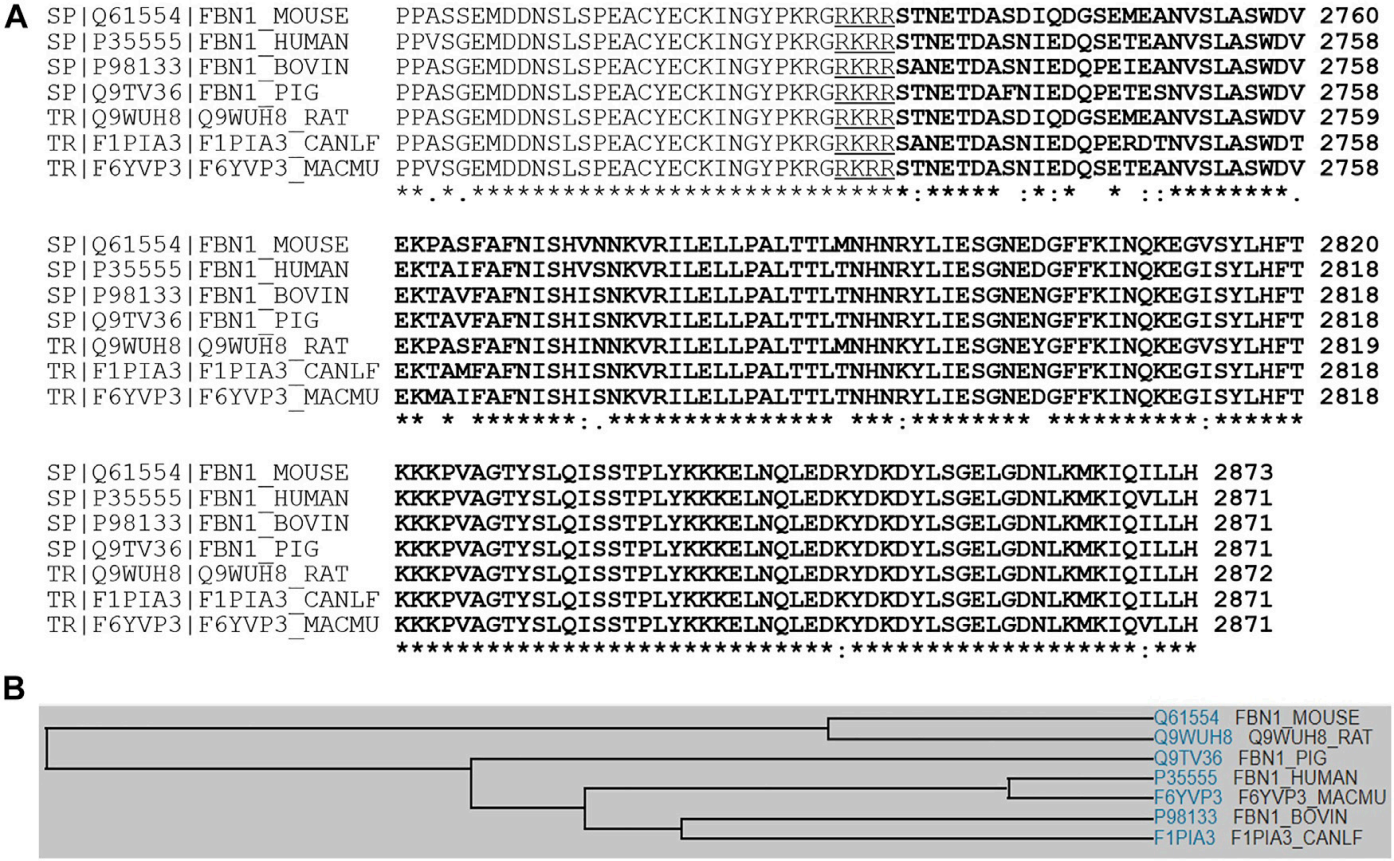
**(A)** Multiple sequence alignment analyses signifying amino acid sequence identity and similarity among asprosin in various species of human, mouse, rat, pig, dog, bovine, and rhesus macaque. **(B)** A family tree of asprosin proteins is presented.

**FIGURE 2:**
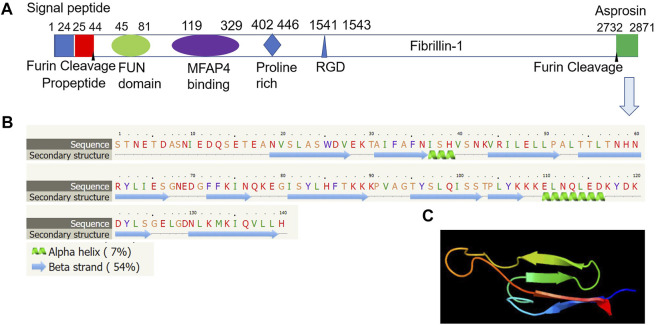
Molecular structure of human asprosin protein. **(A)** Asprosin (aa2732-2871) is derived from the C-terminal region of prefibrillin-1 which contains a signal peptide (aa1-24), propeptide (aa25-44), a furin cleavage motif after RAKR (aa44), fibrillin unique N-terminal (FUN) domain (aa45-81), microfibrillar-associated protein 4 (MFAP4) interacting domain (aa119-329), proline rich domain (aa402-446), RGD motif (aa1541-1543), a furin cleavage motif after RKRR (aa2731), and fribrillin-1 (aa25-2731). **(B)** Asprosin is predicted to consist of two alpha helixes and several beta strands based on the analysis by Phyre2 web portal (http://www.sbg.bio.ic.ac.uk/phyre2/). **(C)** Tertiary structure analysis showing human asprosin protein that mimics crystal structure of cadherin8 ec1 domain based on template c1zxkB, which has 46 residues (33% of asprosin sequence) have been modelled with 95.2% confidence by the single highest scoring template using Phyre2 web portal analysis (http://www.sbg.bio.ic.ac.uk/phyre2/).

**FIGURE 3:**
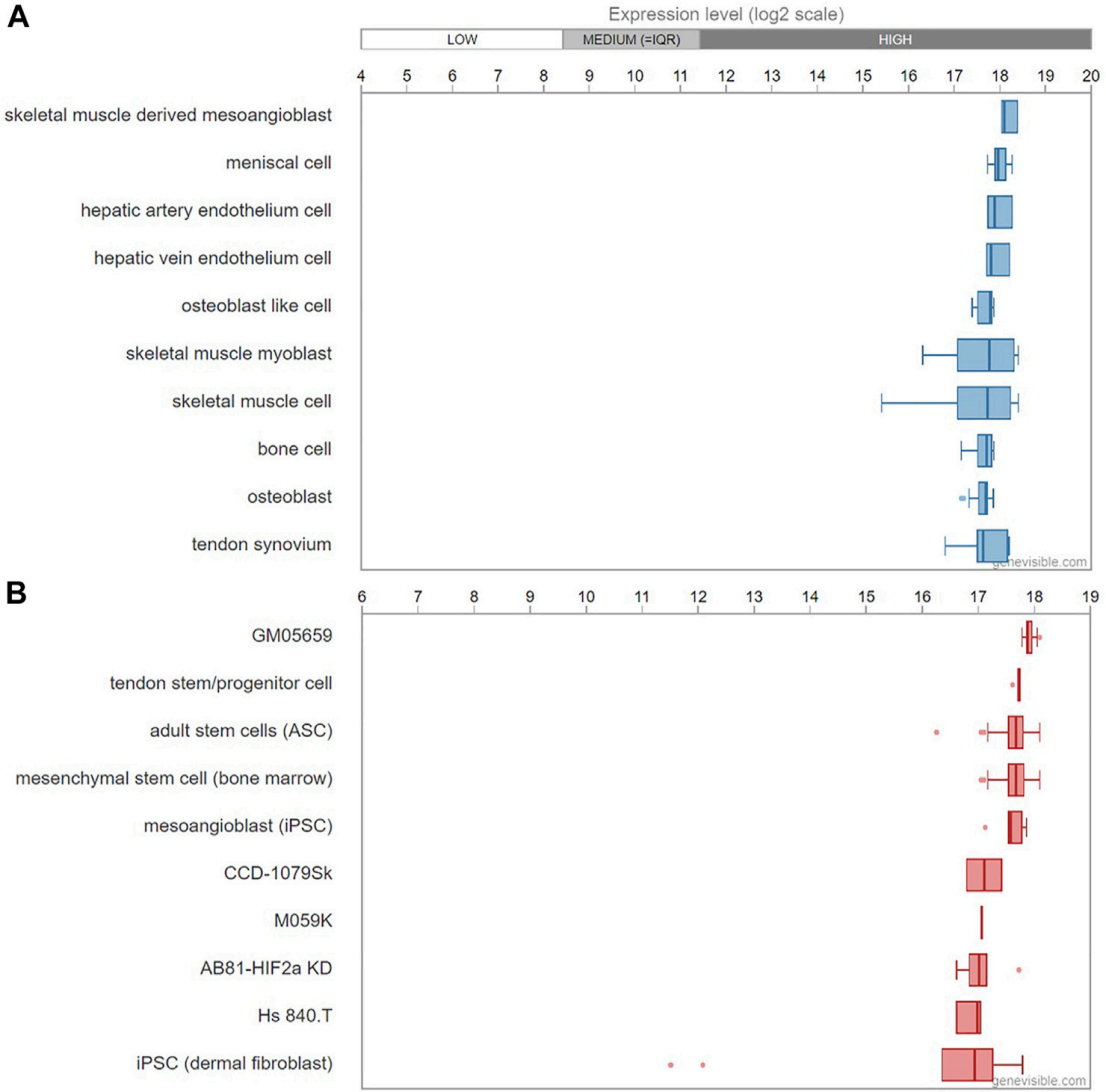
mRNA expression profiling of *FBN1* gene in human tissues **(A)** and cell lines **(B)** predicted by Genevisible^®^ bioinformatics analyses (http://genevisible.com). The ten most highly ranking tissues and cell lines that express *FBN1* mRNA are shown.

Asprosin is encoded by *FBN1* gene and belongs to a post-translationally modified product of fibrillin. **Asprosin is released as the C-terminal propeptide from profibrillin-1, and is cleaved by pro proteinase furin** (Jensen et al., 2014). It is well conserved between human and mouse based on multiple sequence alignment (Figure 1). Molecular structure analysis of human asprosin protein shows that it contains Asprosin (aa2732-2871) at the C-terminal region of prefibrillin-1 which contains a signal peptide (aa1-24), propeptide (aa25-44), a furin cleavage motif after RAKR (aa44), fibrillin unique N-terminal (FUN) domain (aa45-81), microfibrillar-associated protein 4 (MFAP4) interacting domain (aa119-329), proline rich domain (aa402-446), RGD motif (aa1541-1543), a furin cleavage motif after RKRR (aa2731), and fribrillin-1 (aa25-2731) (Figure 2A). Further, Asprosin is predicted to consist of two alpha helixes and several beta strands based on the analysis of the Phyre2 web portal (Figure 2B). Tertiary structure analysis shows that human asprosin protein mimics the crystal structure of cadherin8 ec1 domain using the Phyre2 web portal (Figure 2C). *FBN1* mRNA expression was detected widely in human tissues and cell lines (Figure 3). Gene expression analyses by Genevisible^®^- based bioinformatics (Hruz et al., 2008) reveal that *FBN1* mRNA was most abundantly expressed in human tissues of skeletal muscle-derived mesoangioblasts, meniscal cells, liver artery endothelium cells, liver vein endothelium cells and osteoblast-like cells (Figure 3A). It was most highly expressed in human fibroblast cell lines of GM05659, tendon stem/progenitor cell, adult stem cells, mesenchymal stem cell, and mesoangioblast (Figure 3B). These results indicate that asprosin is likely to be released from these tissues and cell sources. The expression of asprosin varies between different tissues in pathological conditions. For instance, the asprosin level is elevated in gastric and testicular tissues but decreased in liver, kidney, and heart tissues in diabetic rats compared with normal controls (Kocaman and Kuloglu, 2020). Asprosin can circulate to the liver to induce a glucogenic effect through OLFR734, an olfactory G-protein-coupled receptor in liver cells to regulate glucose homeostasis (Li et al., 2019). It also crosses the blood-brain barrier to induce an orexigenic effect via regulating appetite-modulating neurons in the arcuate nucleus of the hypothalamus (Duerrschmid et al., 2017).

During diverse progressions of cell metabolism, asprosin appears to play varying roles. For example, it is shown that asprosin inhibits apoptosis and reactive oxygen species (ROS) generation, through the activation of the ERK1/2-SOD2 pathway, which **increases** the antioxidant level of mesenchymal stromal cells (MSCs) in an ischemic heart disease model. But there is no influence on cell migration and proliferation by asprosin (Zhang Z. et al., 2019).

## Asprosin in Metabolic Diseases

The endocrine system acts through hormones in the regulation of carbohydrate, protein, and fat metabolism, and maintains the development and functional activities of our body. The level of circulating asprosin is positively correlated with risk factors of abnormal metabolism, such as fasting glucose and atherogenesis index in menopausal women (Wiecek et al., 2019). Asprosin is shown to mediate lipid metabolism, which negatively regulates fat browning by reducing the expression of the browning marker, uncoupling protein 1 (UCP1), and by promoting lipid deposition by suppressing nuclear factor E2-related factor 2 (Nrf2) activation, in both a cold-stimulated and high-fat diet murine model (Miao et al., 2021).

## DM

Type 1 diabetes mellitus (T1DM), Type 2 diabetes mellitus (T2DM), and gestational diabetes mellitus (GDM) are three subtypes of DM. Serum asprosin is reported to increase in all these types (Baykus et al., 2019; Ko et al., 2019; Zhang L. et al., 2019). Oral glucose tolerance test (OGTT) is used to assess dynamic changes in insulin secretion following oral glucose load. In normal glucose tolerance (NGT) participants, after a 75 g OGTT, asprosin is negatively correlated with blood glucose (BG) and C-peptide, after adjusting for age, gender, and BMI (Zhang X. et al., 2020). Conversely, in T2DM patients, plasma asprosin level is positively correlated with fasting blood glucose (FBG), hemoglobin A1c (HbA1c), post-challenge plasma glucose (2hPG), homeostasis model assessment for insulin resistance (HOMA-IR), triglyceride (TG), triacylglycerol (TAG) and waist circumference (Wc). Among them, FBG and HOMA-IR are independently correlated with asprosin in T2DM, which was obtained by multiple stepwise regression analysis (Li et al., 2018; Naiemian et al., 2020; Wang et al., 2018). Similarly, plasma asprosin is elevated in GDM patients, as related factors of insulin resistance are increased in the early stages of gestation (Zhong et al., 2020).

The prophase of T2DM, also known as impaired glucose regulation (IGR), including impaired glucose tolerance (IGT) and impaired fasting glycaemia (IFG) (Alberti and Zimmet, 1998), is associated with insulin resistance and β-cell dysfunction (Williamson, 2018), and has a high risk of DM (Tabak et al., 2012). Individuals with IGR show relatively higher plasma asprosin levels than newly T2DM patients, and both are higher than non-T2DM subjects. Subsequent correlation analysis elucidates that plasma asprosin is negatively correlated with a homeostasis model assessment for β-cell function (HOMA-β), acute insulin response (AIR), and glucose disposition index (GDI). Specifically, circulation asprosin level is correlated with IGR and newly T2DM by multiple logistical regression analyses (Wang et al., 2018). In newly diagnosed DM, circulating asprosin is significantly suppressed, after 24-weeks treatment, as well as HbA1c, FPG, and BMI, by sodium-glucose co-transporter-2 (SGLT2) inhibitors, a hypoglycemic agent, which selectively inhibit glucose reabsorption (Jiang et al., 2021). Asprosin therefore appears to be a potential **biomarker** for DM diagnosis.

As diabetes progresses, various complications gradually emerge. In DM patients with kidney disease, circulating asprosin is higher than that in individuals without diabetic nephropathy. Furthermore, patients with macroalbuminuria and microalbuminuria have a significantly elevated asprosin level. Moreover, asprosin level is positively associated with disease duration, urinary albumin-to-creatinine ratio (UACR), creatinine, blood urea nitrogen (BUN), and negatively correlated with estimated glomerular filtration rate (eGFR), and the treatment of metformin, and acarbose, especially in the early stage of nephropathy. Furthermore, asprosin is independently related to UACR, BUN, low-density lipoprotein (LDL)-C, and the development of albuminuria in T2DM patients by regression analyses (Deng et al., 2020; Wang et al., 2021; Zhang H. et al., 2020). Similarly, in diabetic retinopathy (DRP) individuals, the asprosin level is higher compared with non-DRP patients in blood and aqueous humor (Oruc et al., 2020). Collectively, asprosin appears to play a role in the onset and progression of DM and might be a potential therapeutic target for the treatment of this illness.

The researchers have explored the pathogenic mechanism of asprosin in DM. *In vivo* experiments suggest that asprosin could only elevate BG level in normoglycemic mice but not in diabetic ones. Further, intraperitoneal injection of asprosin was found to decrease excessive hepatic TG, cholesterol, and LDL of diabetic animals (Hekim et al., 2021). *In vitro* findings indicated that asprosin could affect the physiological function of insulin-releasing pancreatic β-cells. For example, Lee et al. (2019) examined the knockdown effects of asprosin and its downstream toll-like receptor (TLR) 4 or JNK expressions in a pancreatic β-cell line MIN6. The results demonstrate that asprosin increases the level of inflammatory cytokines TNF and MCP-1, and phosphorylation of nuclear factor-kappa B (NF-κB) to exacerbate inflammation. Analogously, cell viability and insulin secretion are all attenuated via asprosin treatment, through the TLR4/JNK signal pathway. Similarly, research on MIN6 cells reports that recombinant asprosin inhibits the expression of autophagy marker LC3-II/LC3-I and beclin 1, and promotes apoptosis through an AMP-activated protein kinase (AMPK)-mTOR pathway in β-cells (Wang and Hu, 2021).

In addition to disrupting β-cells secretion, asprosin also contributes to insulin resistance. The maintenance of blood glucose homeostasis depends on the insulin sensitivity of tissues such as muscle, liver, and fat. When the extracellular environment is disturbed, increased cellular stress in these tissues hinders the metabolic activity of insulin, resulting in insulin resistance, which is a major determinant of T2DM progression (DeFronzo and Tripathy, 2009; Petersen and Shulman, 2018; Fazakerley et al., 2019). In a normoglycemic hyperinsulinemic model, skeletal muscle takes up about 80% of the total glucose metabolism (DeFronzo et al., 1981), while in a study of leg muscle glucose uptake, insulin-stimulated glucose uptake was reduced by approximately 50% in T2DM. This suggested that skeletal muscle insulin resistance is considered the incipient metabolic disorder of T2DM (DeFronzo et al., 1981; DeFronzo and Tripathy, 2009). Specifically, impaired glycogen synthesis is a major mechanism leading to muscle insulin resistance. During the process of insulin-mediated glucose uptake, it has been suggested that the IRS-1/PI-3 kinase/Akt pathway is critical (Krook et al., 2000; DeFronzo and Tripathy, 2009). However, the regulatory role of asprosin in this pathway remains to be elucidated. An intervention with asprosin in myocyte cell line C2C12 and soleus skeletal muscle reveals that asprosin accelerates insulin resistance by stimulating the ER stress/inflammation-mediated pathway. In addition, asprosin suppresses the expression of protein kinase C-δ (PKCδ) phosphorylation by high-affinity binding and inhibits sarcoplasmic reticulum Ca^2+^ ATPase 2b level, resulting in impaired myocyte insulin sensitivity (Jung et al., 2019).

### Obesity

Obesity, especially abdominal obesity, is often accompanied by metabolic disorders such as hyperglycemia and dyslipidemia, and cardiovascular disease morbidity and mortality (Engin, 2017). In obese subjects, circulating asprosin is not only increased compared with normal weight but also elevated to accompany the rising BMI, in both adults and children (Ugur and Aydin, 2019; Sunnetci Silistre and Hatipogl, 2020). Analogously, in underweight subjects and weight reduction after bariatric surgery participants, asprosin levels are also associated with BMI (Ugur and Aydin, 2019; Wang C. Y. et al., 2019). The studies of obesity in children reveal that asprosin level is increased in children with insulin resistance as compared with the non-insulin resistance group. Among obesity-related indicators, waist-to-hip ratio (WHR) and HOMA-IR are positively associated with asprosin, which is considered to be a predictor of obesity by multiple regression analysis (Wang M. et al., 2019; Sunnetci Silistre and Hatipogl, 2020).

Additional research of asprosin regulation in an obesity model of monosodium glutamate (MSG)-induced hypothalamic obesity mice showed that 3-weeks treatment by AM6545, a peripheral cannabinoid receptor 1 (CB1R) blocker, diminishes the level of asprosin while reducing the increased body weight, dyslipidemia and intraperitoneal fat mass (Ma et al., 2018). Irisin, a peptide hormone secreted by skeletal muscle, also regulates asprosin expression. Ozcan et al. (2020) reported that asprosin level is decreased in female obese rats with irisin subcutaneous administration, as well as excessive blood glucose, LDL, and TG.

For obesity, appetite is an effective predictor of reduced energy intake and weight loss (Drapeau et al., 2007). **Duerrschmid et al. found that decreasing the plasma asprosin level by monoclonal antibody reduces both appetite and body weight in obese mice to ameliorate BG level.** This result is due to the appetite-regulating effect of asprosin, which promotes the activity of orexigenic receptor protein tyrosine phosphatase receptor δ (Ptprd) in AgRP^+^ neurons in a cAMP-dependent manner; whilst inhibiting anorexic POMC^+^ neurons in a GABA-dependent pathway, thereby stimulating appetite and promoting bodyweight accumulation similar to ghrelin (Duerrschmid et al., 2017; Donma & Donma, 2018; Mishra et al., 2022).

## PCOS

Polycystic ovary syndrome (PCOS) is a gynaecological syndrome with characteristics of hyperandrogenemia, ovulatory dysfunction, and/or ovarian polycystic pathology. PCOS patients have metabolic disorders, such as insulin resistance and dyslipidemia (Deniz et al., 2021). Asprosin levels are found to be higher in PCOS patients, and in addition, individuals with the highest asprosin secretion have the greatest risks of PCOS (Alan et al., 2019). Plasma asprosin **concentration** is an independent risk factor for PCOS, according to the binary logistic regression analysis (Li et al., 2018). Similar to T2DM, asprosin is reported to be positively associated with FBG, HbA1c, HOMA-IR, and testosterone in PCOS (Deniz et al., 2021; Li et al., 2018). Asprosin could therefore become a novel metabolic marker of PCOS.

In other endocrine diseases, studies have found that asprosin is involved in different pathological processes. Non-alcoholic fatty liver disease (NAFLD) is a type of hepatic disease, which is associated with insulin resistance, metabolic syndrome, and type 2 diabetes (Cobbina and Akhlaghi, 2017). In NAFLD patients, serum asprosin is higher than in healthy individuals, and is independently related to FBG and HOMA-IR by multivariate linear regression analyses (Ke et al., 2020).

## Asprosin in Exercise-Regulating Metabolic Diseases

Exercise is essential for human health, especially for the endocrine system. Lack of exercise and obesity are attributed to chronic oxidative stress, which leads to instability of insulin secretion and vascular complications. Physical exercise could induce adaptive responses to maintain the redox balance, thereby controlling disease progression and complications (Poblete-Aro et al., 2018). Meta-analysis shows that structured exercise intervention is effective for glycaemic control in T2DM patients with decreased insulin resistance (Sampath Kumar et al., 2019).

Exercise affects the expression of asprosin, such that untrained women have higher circulating asprosin concentrations during the menstrual cycle **especially in the follicular phase**, compared with trained women (Leonard et al., 2021). A single short-term anaerobic exercise for a 20s bicycle sprint indicated that both circulating asprosin and BG are elevated after exercise, interestingly, this influence only appears in women, and the mechanism remains unknown (Wiecek et al., 2018). In addition, exercise also plays a different role in diverse metabolic diseases through asprosin.

In DM, appropriate exercise ameliorates hyperglycemia by regulating hepatic glucose metabolism. Asprosin is reported to increase the release of hepatocyte glucose via a cAMP-PKA axis, independent of glucagon or epinephrine (Romere et al., 2016). While, an eight-week aerobic exercise decreases the hepatic asprosin level by PKA/TGF-β pathway, and increases the AMPK-related signal pathway to alleviate impaired glucose metabolism and help with DM treatment, in a study of streptozotocin (STZ)-induced diabetic rats (Ko et al., 2019). Additionally, exercise also accelerates insulin sensitivity and glycogen storage of skeletal muscle by increasing glucose transporter 4 (GLUT4) expression (Holloszy, 2005; Richter and Hargreaves, 2013). Conversely, a recent study of skeletal muscle glucose uptake indicates that asprosin has three glycosylation sites and enhances glucose transport by elevating the level of GLUT4 in myotubes. Further, the glucose uptake is promoted by AMPK phosphorylation in skeletal muscle by exogenously administering asprosin (Zhang et al., 2021). These findings contradict other studies considering the differential effects of asprosin on various pathways, which deserves further research. Notably, different forms of exercise have diverse effects on DM improvement. A meta-analysis shows that both aerobic and resistance exercise are effective for the reduction of HbA1c, but combined exercise generates more improvement than either aerobic or resistance exercise (Pan et al., 2018).

In obesity, regular physical activity reduces adipose tissue and weight and improves fat browning by increasing UCP1 (Reisi et al., 2016; Mu et al., 2021). During exercise, triacylglycerols, an adipose tissue energy metabolite, are hydrolyzed into free fatty acids (FA), which are then released into the circulation to empower the muscle to function (Mika et al., 2019). After moderate intensity aerobic exercises, serum asprosin is significantly decreased in overweight and obese participants (Ceylan et al., 2020).

In PCOS, a previous meta-analysis concludes that vigorous-intensity exercise accelerates the indicators such as VO2peak, BMI, and waist circumference in patients, thereby improving cardiopulmonary function, insulin sensitivity, and ovulation function (Hakimi and Cameron, 2017; Patten et al., 2020). Asprosin is reported to downregulate mTOR expression, which inhibits follicular activation in PCOS (Wang and Hu, 2021; Zhang et al., 2022), while exercise decreases asprosin level and improves mTOR concentration (Ko et al., 2019; Stepto et al., 2020), so it is speculated that asprosin might play a role in alleviating PCOS with exercise.

Both NAFLD and thyroid function benefit from exercise (Farzanegi et al., 2019; Masaki et al., 2019). For instance, exercise enhances lipophagy to diminish liver steatosis by stimulating the AMPK/SIRT1 pathway (Li et al., 2021). In prophyltiouracil (PTU) induced hypothyroidism rats, asprosin level is lower than the control group, and after thyroxine treatment, asprosin is elevated (Mogulkoc et al., 2020). However, the role of asprosin in these conditions requires further investigation.

## Summary

**FIGURE 4:**
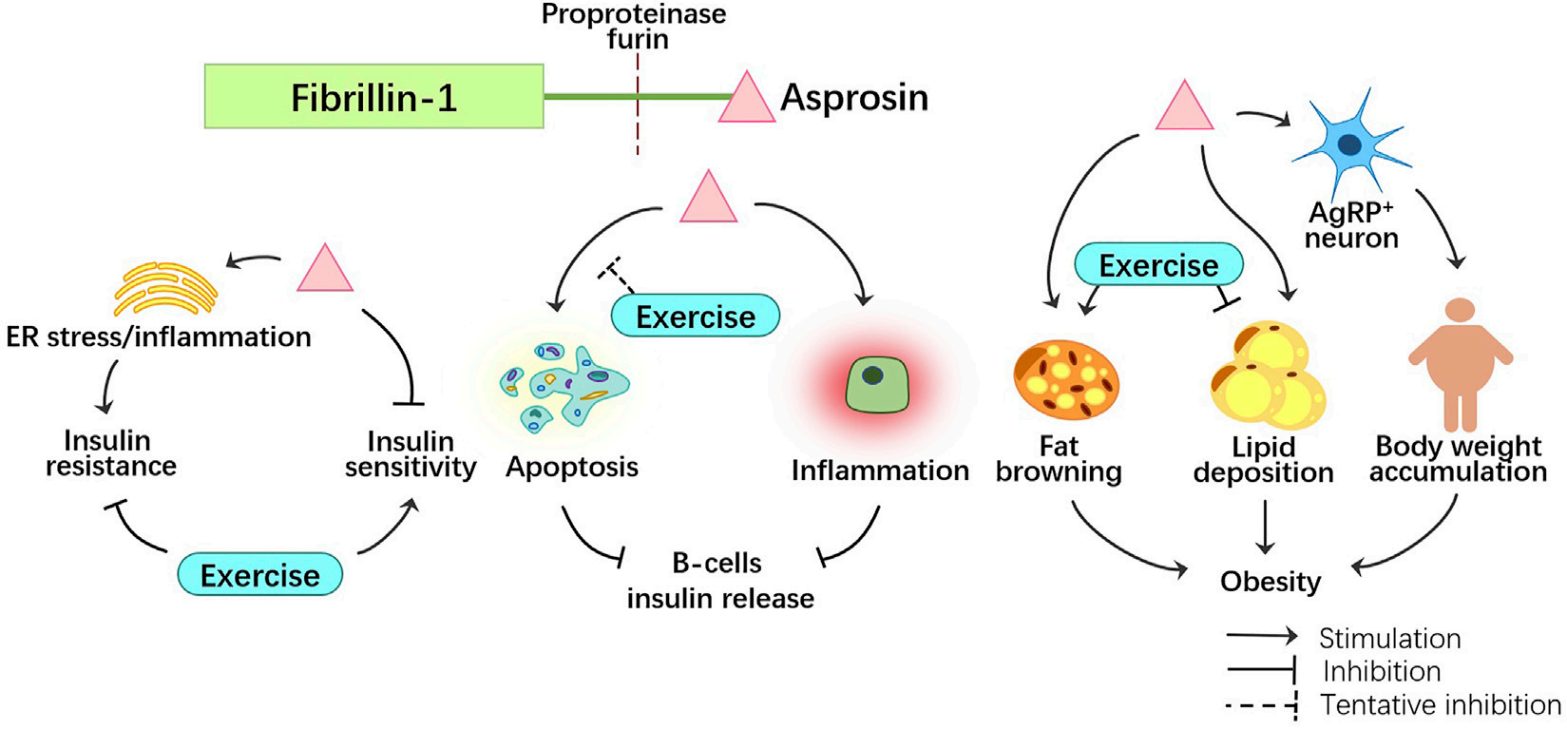
The role of asprosin in metabolic disorders, such as insulin resistance, decreased insulin release and obesity. Exercise plays an inhibitory role to asprosin, helping to mitigate metabolic disorders.

As a new protein discovered recently, asprosin has attracted great interest for its role in metabolism and diseases. In metabolic disorders such as DM, obesity, and PCOS, asprosin could serve as a biomarker for early diagnosis and a therapeutic target. Exercise, as an effective intervention for regulating metabolism and endocrine activity, is a preferred non-pharmacological therapy in many metabolic diseases. The regulation of asprosin on metabolic abnormalities and the important role of exercise are summarized in Figure 4. Asprosin has a vital regulatory effect on the improvement of metabolic diseases by exercise and is expected to become an important target of exercise regulation in future scientific research and clinical practice.
